# Susac syndrome in a patient with migraine shortly after COVID-19 booster vaccination: more than a temporal relation?

**DOI:** 10.1007/s00415-023-12088-0

**Published:** 2023-11-09

**Authors:** Stergios Tsitos, Adrian Danek, Andreas Straube

**Affiliations:** Department of Neurology, University Hospital, Ludwig-Maximilians-Universität (LMU), Munich, Germany

Dear editors,

Susac syndrome is a rare, immune-mediated endotheliopathy with a classical triad of encephalopathy, branch retinal artery occlusions and sensorineural hearing loss. Since first described in 1979, around 400 cases have been reported [1]. Headache, most commonly migraine-like, is the most common symptom, occurring in around 80% of the cases. On MRI, small ischemic lesions are seen, typically in the corpus callosum, leptomeninges, brainstem, cerebellum and cerebellar peduncles [2]. Histopathological examination of the lesions shows infiltration of dysregulated CD8 + T-lymphocytes into the lumen and perivascular space of cerebral microvessels and direct apposition of these lymphocytes to apoptotic endothelial cells, interacting with their major histocompatibility complex class I (MHC-I) [3]. Blood and CSF analyses show oligoclonal expansion of terminally differentiated CD8 + T-lymphocytes. Treatment consists of immunosuppression, almost always initiated by a pulse of corticoids and usually accompanied by further immunosuppressive treatments. Given the recent insight into the pathophysiology of Susac syndrome, Natalizumab has been proposed as a treatment option, impeding interaction between CD8 + T-cells and endothelial cells and has been deemed successful in a series of patients [3].

Migraine is one of the most common primary headache syndromes, affecting around 16% of the general population. Its pathophysiology has not been fully understood yet. The significance of the trigeminovascular system, however, has been highlighted on multiple occasions [4]. Blood vessels seem to possess the highest density of unmyelinated C-fibers and thinly myelinated Aδ-fibres within meninges and stimulation of large meningeal vessels is associated with headache [4]. Activation of these nerve endings and signal transmission to the thalamus and thereafter the cerebral cortex is considered as the pathophysiological equivalent of pain. Recently, the potential role of neuroinflammation in this process has been highlighted. Byproducts of inflammatory processes, such as arachidonic acid and potassium, have been shown to be able to activate these perivascular nerve endings and cause further neurogenic inflammation [5]. Furthermore, animal models have shown that cortical spreading depressions, considered to be the pathophysiological equivalent of migrainous aura that typically precedes headache, can cause a proinflammatory state by activation of Pannexin 1 mega channels and release of high-mobility group box 1 (HMGB1) from neurons, leading to a prolonged inflammatory response of glial cells by activation of the NFκB pathway and thereafter the NLRP3 inflammasome and release of IL-1ß [6]. This inflammatory milieu can then cause activation of the trigeminovascular system, ultimately leading to headache.

We present the case of a 33-year-old female that presented in our emergency department after having developed multiple neurological symptoms over the course of almost 2 months. The patient had a past medical history of migraine without aura, with a frequency of around two episodes per year, well controlled with triptans. Her family history was not significant for neurological or autoimmune conditions. Two days after receiving her booster vaccination against SARS-CoV-2, she experienced headache of the same quality as her general migraine, but unusual lasting 5–6 days and not responding to her usual medication. Around one week later, she experienced scintillating scotomas lasting up to one hour and recurring three to four times daily. She presented to the emergency department of an external hospital, where she was discharged from with the presumed diagnosis of a migraine with aura, given her past medical history. Around three weeks after this event, she experienced numbness from her lower chest downwards. She was admitted to an external hospital but was discharged after unremarkable results on MRI and CSF analysis. One week later, she experienced right-sided hearing loss. An outpatient otorhinolaryngology specialist diagnosed acute hearing loss and prescribed oral prednisolone (initial dose 1 mg/kg, tapering over 2 weeks). Another week later, she experienced progressive vertigo, tiredness and general weakness, leading to presentation in our emergency department.

Upon neurological examination, she showed a spontaneous nystagmus to the left, a left-sided hemiataxia and atactic gait. Audiometry showed low-frequency hearing loss on the right ear (Fig. 1), electroencephalography showed mild diffuse encephalopathy. CSF analysis showed no pleocytosis, but mild disturbance of the blood–brain barrier. No oligoclonal bands were found. Cranial MRI showed multiple diffusion-restricted lesions in cortical and subcortical localization involving the corpus callosum and the left cerebellar peduncle (Fig. 2). Gadolinium enhanced images were also obtained showing gadolinium uptake in almost half of the lesions. Spinal MRI showed a lesion affecting the dorsal columns at the height of the second cervical vertebra. Retinal fluorescence angiography showed multiple branch retinal artery occlusions, thus complying the diagnostic criteria for Susac syndrome proposed by Kleffner et al. [7]. Analysis of lymphocytes in the peripheral blood showed an increased proportion of activated cytotoxic T-cells in accordance with their assumed importance in the pathogenesis of the disease. Transthoracic echocardiography showed no cardiac source of embolism. Transesophageal echocardiography was not tolerated by the patient. Base coagulation studies (INR, PTT, platelet count) were unremarkable. Studies for antiphospholipid syndrome (lupus anticoagulant, cardiolipin and ß2-glycoprotein antibodies) also yielded no positive result. Imaging of the vessels supplying the brain (via CT-angiography, TOF-MRA and duplex sonography) also showed no signs of macroangiopathy. A corticoid pulse and plasma exchange led to prompt clinical improvement and the patient was discharged with oral steroids and under Natalizumab. During the following months, the patient experienced relapses, presenting with migrainous headaches, vertigo and new lesions on MRI making corticoid pulses and temporary increase of the dosage of oral steroids necessary. Because of suboptimal disease control under Natalizumab and elevated JC-virus antibody index, the patient was switched to intravenous immunoglobulins and mycophenolate 6 months later. The patient has since been well controlled and able to taper the dose of corticoids below the Cushing threshold.

**Fig. 1:**
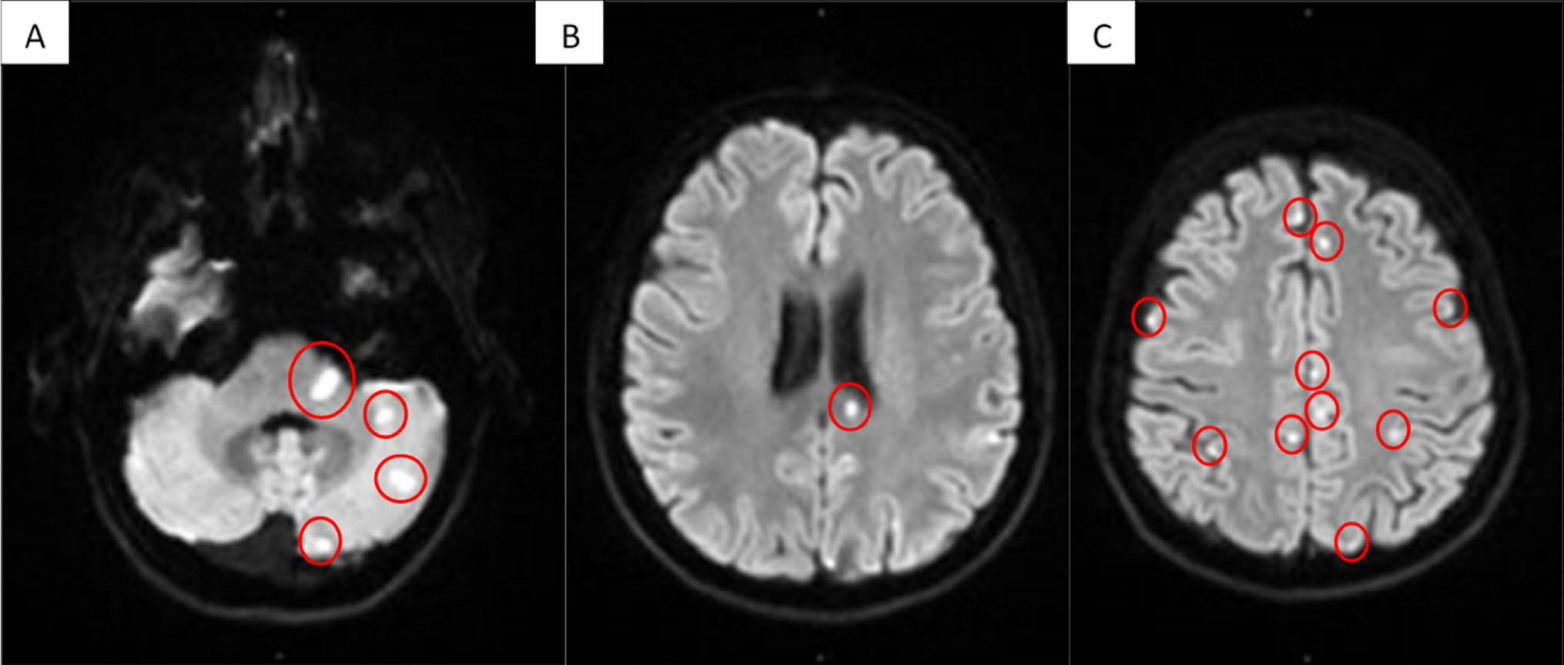
Typical findings of Susac syndrome. Diffusion weighed imaging (DWI) lesions of the left middle cerebellar peduncle and cerebellar hemisphere (**a**), the corpus callosum splenium (**b**), as well as in multiple cortical and subcortical localisations (**c**) on MRI

**Fig. 2:**
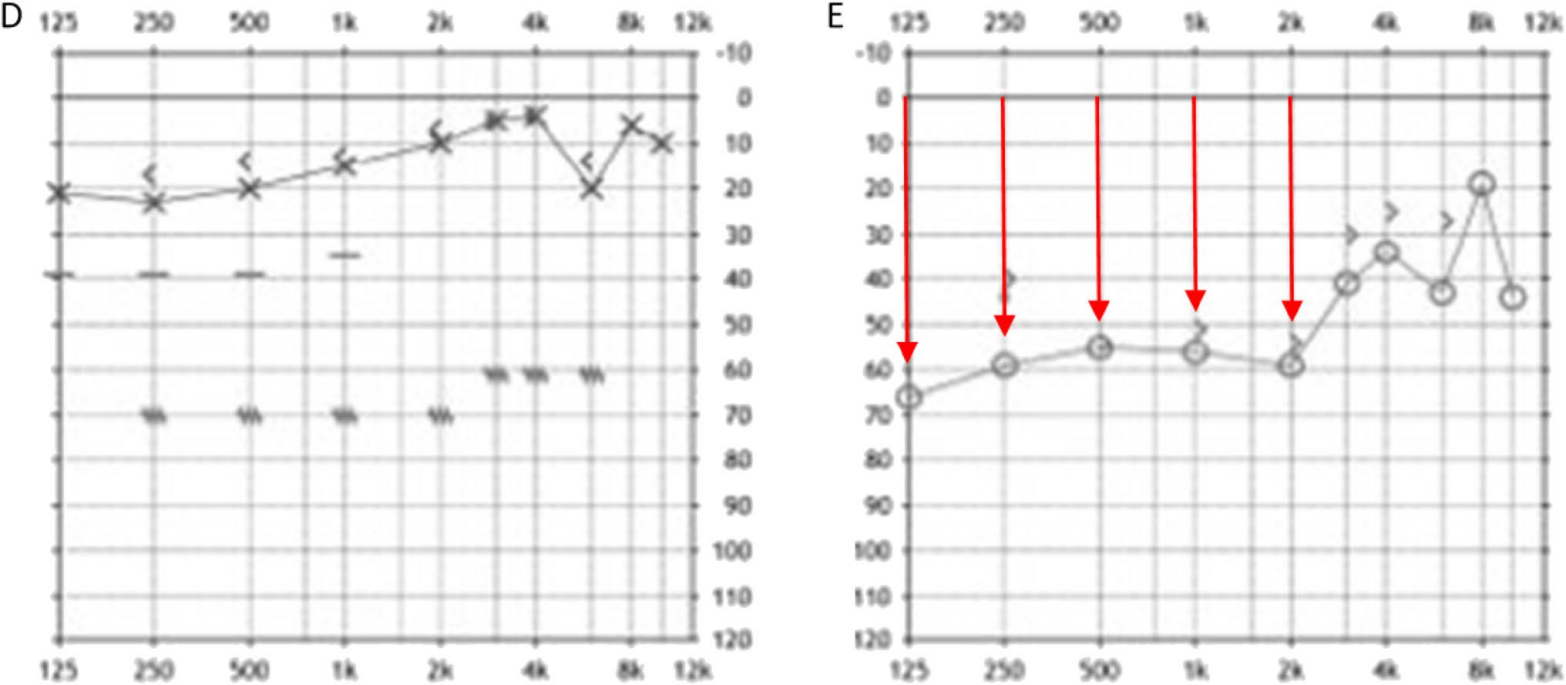
Results of audiometry, showing sensorineural hearing loss pronounced in lower frequencies on the right ear (**e**) and normal result on the right ear (**d**)

Susac syndrome is a rare CD8-T-cell-mediated endotheliopathy affecting the central nervous system, inner ear and retina. Here, we present a case where the disease presumably was triggered by a booster immunization against SARS-CoV-2 and initially manifested with headache of the same quality the patient was familiar with from her migraine.

CD8-T-cell-mediated cytotoxicity through cell lysis has long been considered to be a sterile process. However, recent data from murine models studying dysregulated CD8 + T cell cytotoxicity in other conditions have shown its ability to produce chronic inflammation by activation of NLRP3 inflammasome and production of IL-1ß and other inflammatory cytokines [8]. The same pathway has been shown to be activated through cortical spreading depressions, the pathophysiological equivalent of auras, and has been linked to activation of the trigeminovascular system and thereafter headache in migraine [6]. Neuroinflammatory processes and activation of the NLRP3 inflammasome in the meninges seem, therefore, to play a central role in leading up to and sustaining migrainous headache in both Susac syndrome and migraine itself either through CD8-T-cell-mediated cytotoxicity in the first case or through cortical spreading depressions or other ways of activation of pseudounipolar cells of the trigeminal ganglion in the latter.

Interestingly, especially after secondary immunisation, like in our patient, animal model of SARS-CoV-2 vaccination has shown activated CD8 + T-cells to play an important role in the immune response within the first hours after immunisation by producing proinflammatory cytokines like IFN-γ and tumor-necrosis factor (TNF), which in turn have been shown to cause inflammasome-mediated endothelial cell death [9].

Furthermore, there is increasing evidence showing that in headaches related to SARS-CoV-2-infection NLRP3 plasma levels are increased [9]. The Bruton tyrosin kinase is part of the NLRP3-inflammasome and the inhibition due to specific inhibitors (e.g. ibrutinib) improves the outcome in patients with severe SARS-CoV-2 infection [10]. Up to now, no study about the effect of such an inhibitor on post-COVID headache has been published but it was experimentally shown that the inhibition of the inflammasome activation is able to reduce the damage in ischemic brain injury [11].

This highlights the importance of neuroinflammation as a possibly targetable pathway in the pathophysiology of the Susac syndrome as well as migraine and the activation of these pathways by SARS-CoV-2 infection or vaccination, presumably as an immunological reaction to the spike protein could be one trigger for these disorders. More studies are needed to assess the role of the NLRP3 activation in these disorders and to evaluate the potential value as a therapeutical target in migraine patients as well as in Susac syndrome.

Finally, we would like to highlight the importance of imaging in a patient with a known migraine when there is a change in character of the headache, like in this case, to avoid missing out on potentially threatening differential diagnoses.

## Data Availability

The participants of this study did not give written consent for their data to be shared publicly, so due to the sensitive nature of the research supporting data is not available.
